# Efficacy, Safety, and Cost-Minimization Analysis of Continuous Infusion of Low-Dose Gemcitabine Plus Cisplatin in Patients With Unresectable Malignant Pleural Mesothelioma

**DOI:** 10.3389/fonc.2021.641975

**Published:** 2021-04-20

**Authors:** Oscar Arrieta, Wendy Muñoz-Montaño, Sae Muñiz-Hernández, Saul Campos, Rodrigo Catalán, Herman Soto-Molina, Silvia Guzmán Vázquez, Osvaldo Díaz-Álvarez, Victor Martínez-Pacheco, Jenny G. Turcott, Maritza Ramos-Ramírez, Luis Cabrera-Miranda, Feliciano Barrón, Andrés F. Cardona

**Affiliations:** ^1^ Laboratory of Personalized Medicine, Instituto Nacional de Cancerología, Mexico City, Mexico; ^2^ Thoracic Oncology Unit, Instituto Nacional de Cancerologia, Mexico City, Mexico; ^3^ Department of Medical Oncology, ISSEMYM, Toluca, Mexico; ^4^ Department of Economic Evaluation, AMETSA, Mexico City, Mexico; ^5^ Foundation for Clinical and Applied Cancer Research (FICMAC), Bogotá, Colombia; ^6^ Clinical and Translational Oncology Group, Clinica del Country, Bogota, Colombia

**Keywords:** pleural mesothelioma, prolonged gemcitabine infusion, low-dose gemcitabine, cisplatin, chemotherapy, cost minimization analysis

## Abstract

**Background:**

Malignant pleural mesothelioma (MPM) is rare and aggressive neoplasia, with a poor prognosis; furthermore, the monetary cost of its treatment represents a major challenge for many patients. The economic burden this malignancy imposes is underscored by the fact that asbestos exposure, which is the most frequent risk factor, is much more prevalent in the lower socioeconomic population of developing countries. The aims of the present study were to evaluate the efficacy, safety, and cost of continuous infusion of low-dose Gemcitabine plus Cisplatin (CIGC) as a treatment strategy for patients with unresectable MPM.

**Methods:**

We performed a prospective cohort study to determine efficacy and safety of continuous infusion gemcitabine at a dose of 250 mg/m2 in a 6-h continuous infusion plus cisplatin 35 mg/m2 on days 1 and 8 of a 21-day cycle in patients with unresectable MPM. We also performed a cost-minimization analysis to determine if this chemotherapy regimen is less expensive than other currently used regimens.

**Results:**

The median number of chemotherapy cycles was six (range 1–11 cycles); objective response rate was documented in 46.2%, and disease control rate was seen in 81.2%. Median PFS was 8.05 months (CI 95% 6.97–9.13); median OS was 16.16 months (CI 95% 12.5–19.9). The cost minimization analysis revealed savings of 66.4, 61.9, and 97.7% comparing CIGC with short-infusion gemcitabine plus cisplatin (SIGC), cisplatin plus pemetrexed (CP), and cisplatin plus pemetrexed and bevacizumab (CPB), respectively. Furthermore, this chemotherapy regimen proved to be safe at the administered dosage.

**Conclusion:**

CIGC is an effective and safe treatment option for patients with unresectable MPM; besides, this combination is a cost-saving option when compared with other frequently used chemotherapy schemes. Therefore, this treatment scheme should be strongly considered for patients with unresectable MPM and limited economic resources.

## Background

Malignant pleural mesothelioma (MPM) is rare and aggressive neoplasia, with a poor prognosis; furthermore, the monetary cost of its treatment represents a major challenge for many patients (1–3). The incidence of this malignancy varies widely among countries; this variation might be explained by the heterogeneous usage of asbestos, which is a well-known risk factor for developing MPM (4). While developed countries have banned the usage of asbestos, in some developing countries, asbestos exposure continues to be relatively frequent (5). Considering that many developed countries banned asbestos usage recently, it is prognosed the incidence of MPM will peak during this decade, owing to the long latency period between asbestos exposure and MPM diagnosis, which might vary between 10 and 40 years (6, 7).

MPM is commonly diagnosed at advanced stages when surgical resection is no longer feasible and palliative therapy is frequently the only option. Even when surgery is successfully performed, recurrence rates are extremely high, reflecting the difficulty of attempting a curative intent approach in patients affected by this neoplasm. It should be underscored that prognosis for patients with MPM have improved marginally during the last decade; therefore, developing new therapeutic approaches with novel and more effective systemic therapies is essential to change the current landscape (8–10). Recently, the FDA approved combination of ipilimumab plus nivolumab as a first line of treatment for patients with unresectable MPM. This approval was based on the result of CheckMate 743 trial, which demonstrated that nivolumab plus ipilimumab significantly extended overall survival *versus* cisplatin plus pemetrexed chemotherapy (median overall survival 18·1 months *vs* 14·1 months]; p = 0·0020) (11).

Currently, the standard therapeutic approach for patients with resectable MPM includes surgery followed by chemotherapy, with or without radiotherapy; however, even with a multidisciplinary approach, the median overall survival (OS) rarely exceeds 18 months, and only 15% of patients remaining alive after five years (12–14). Even in patients who received trimodal treatment, the median OS oscillates from 23 to 29 months, pointing out the dismal prognosis of this disease (15, 16). Prior to approval of ipilimumab/nivolumab as the first line of therapy for patients diagnosed with unresectable disease, chemotherapy with pemetrexed/cisplatin ± bevacizumab was the benchmark therapy (17), yielding a median OS of approximately 18 months and a median progression-free survival (PFS) of 9 months (18–21). Albeit the introduction of immunotherapy as the standard of care for patients with MPM will improve the prognosis of patients with this malignancy, a widespread usage of these drugs should not be expected in developing countries, where costs related to immunotherapy are often not affordable for most patients.

Even before immunotherapy, the economic burden that this malignancy imposes was underscored by the fact that asbestos exposure, which is the most frequent risk factor, is much more prevalent in the lower socioeconomic population of developing countries (22). Indeed, many patients who will be diagnosed with MPM might not be able to afford the associated costs of previous standard first-line treatment based on chemotherapy doublet (cisplatin plus pemetrexed) with or without bevacizumab. Borrelli *et al.* estimated that the total cost of chemotherapy with pemetrexed plus cisplatin in the United States was around USD38,779. In cases suitable to add an antiangiogenic drug like bevacizumab (as recommended for unresectable disease) the chemotherapy regimen costs would increase almost three times (USD87,741) (23, 24), which is considerably exceeds the median annual *per capita* gross domestic product (GDP) in many developing countries.

In 2008 Cordony et al. published a cost-effectiveness analysis of pemetrexed plus cisplatin-based regimen compared with cisplatin monotherapy or MVC (mitomycin C, vinblastine, and cisplatin plus vinorelbine) for the treatment of unresectable MPM; at the analysis, pemetrexed/cisplatin was the best cost-effective treatment option in the United Kingdom. Remarkably, the overall survival under pemetrexed and cisplatin was not impaired despite the additional toxicity seen with the combination (22).

Before pemetrexed became the most common used therapy, the combination of gemcitabine plus cisplatin was one of the most widely used chemotherapy regimens in patients with MPM (25–28). Pooled data from numerous studies in the last three decades, testing gemcitabine and cisplatin combination, led to an estimated OS of 11.7 months for patients with MPM (25–28). In 1997, Pollera et al. demonstrated that gemcitabine retained its antitumor activity at doses as small as 300 mg/m^2^ when given as a prolonged infusion (29). Years after, Kovac *et al.* reported that first-line therapy with a continuous infusion of low-dose gemcitabine plus cisplatin yielded a median PFS of 8 months and median OS of 17 months in patients with unresectable MPM. Moreover, this therapy rendered an acceptable security profile and was associated with an improved quality of life (24). In 2014, our group evaluated the efficacy and toxicity of low-dose gemcitabine in a 6-h continuous infusion plus cisplatin in patients with advanced MPM, in that study 39 patients were evaluated and results were in line with those reported by Kovac et al. The median PFS was 6.9 months, and the median OS was of 20.7 months (1). Based on these results, we established this treatment regimen as a plausible and less expensive alternative in our population.

The aims of the present study were to evaluate the efficacy, safety, and cost of continuous infusion of low-dose Gemcitabine plus Cisplatin (CIGC) as a treatment option for patients with unresectable MPM; and to compare it with other available alternatives such as short infusion of gemcitabine plus cisplatin (SIGC), cisplatin plus pemetrexed (CP), and cisplatin plus pemetrexed and bevacizumab (CPB) from the public and private perspective of our country.

## Methods

### Efficacy and Safety Analysis

Patients with histologically confirmed MPM diagnosis were evaluated by a multidisciplinary team at the Thoracic Oncology Unit of *Instituto Nacional de Cancerologia* *(INCan)* in Mexico City, Mexico. Patients with advanced disease were included in our study in accordance to the predetermined inclusion criteria, which were: ≥18 years old; chemotherapy-naïve unresectable disease; life expectancy ≥3 months; Eastern Cooperative Oncology Group performance status (ECOG) 0–3; adequate renal, hematopoietic, cardiac, and liver function tests results; and willingness to participate and sign the Institutional approved informed consent. Patients were excluded if they presented another malignancy in the previous 5 years. This study was performed in accordance with the Declaration of Helsinki and the principles of good clinical practice. All patients provided written informed consent to participate, and the scientific and bioethical committees of *INCan* approved the entire study.

Before treatment initiation, complete medical history and physical examination including baseline characteristics such as age, gender, asbestos exposure, smoking history, ECOG performance status, Karnofsky Performance Status, European Organization for Research and Treatment of Cancer (EORTC) prognosis group, and Cancer and Leukemia Group B (CALGB) status were obtained. To assess appropriate cardiovascular, renal, liver, and hematologic functions, a complete blood count (CBC), serum chemistry, liver function tests, and creatinine clearance were obtained. A baseline computerized tomography (CT) of chest and abdomen was performed for staging purposes according to the AJCC 8th edition manual. Irresectable disease was determined after discussing each individual patient clinical scenario by a multidisciplinary board including a medical oncologist, a thoracic surgeon, and a radiation oncologist.

All patients received gemcitabine at a dose of 250 mg/m2 in a 6-h continuous infusion plus cisplatin 35 mg/m2 on days 1 and 8 of a 21-day cycle. Patients who were not suitable to receive cisplatin received carboplatin (area under the concentration-time curve 5 mg/ml per min intravenously) once every 3 weeks for up to six cycles.

Treatment was continued until disease progression, unacceptable toxicity, death, or withdrawal from the study. Every patient received prophylactic antiemetic therapy with ondansetron (8 mg), dexamethasone (8 mg), and aprepitant (125 mg) on days 1, 2, 3, 8, 9, and 10.

To classify adverse events (AE), the National Cancer Institute Common Toxicity Criteria (version 4.0) was used to grade treatment toxicity. A dose reduction of up to 20% was allowed for each drug, and treatment delay was permitted if grade 3–4 toxicities were not resolved after 1 week. A CBC, liver function test, and creatinine clearance were obtained on days 1 and 8 of each cycle to assess treatment toxicity.

Response to treatment was classified according to modified Response Evaluation Criteria in Solid Tumors (RECIST) criteria in MPM (30). To evaluate response, a CT-scan was obtained every two cycles of treatment (6 weeks). Patients with response to chemotherapy were reassessed by a multidisciplinary team to determine whether they were eligible for surgical resection; if surgery was not feasible, chemotherapy regimen was continued.

The 30-item EORTC Quality of life (QoL) Questionnaire (EORTC QLQ-C30) version 3.0/Mexican version was used to assess QoL. The EORTC QLQ v3 consists of five multi-item functional scales, three symptom scales, a global health status/QoL scale, and six single items. The transformation of scores was calculated according to the instructions in the manual (31). Scores on all scales and single items range from 0 to 100 points; higher scores on the functional and global health status QoL scales reflect better functioning. For symptom scales, higher scores translate into more severe symptoms. Quality of life questionnaires were filled out 1 day before the first chemotherapy cycle and after two cycles of chemotherapy. We decided to re-evaluate QoL after two cycles in order to correlate it with obtained response assessed on imaging studies (CT-scan); furthermore, by evaluating QoL after two cycles, we were able to asses more patients, since loss of follow-up was minimal after two-cycles.

For statistical analysis, continuous variables were summarized as mean, medians, and standard deviations; categorical variables were summarized as proportions and 95% confidence intervals (95% CIs). The *x* ^2^ or the Fisher exact test were used to determine statistically significant differences among categorical variables. Comparisons between the QoL status were performed before starting treatment, and after the second cycle of chemotherapy, results were analyzed with the Wilcoxon- related samples test. Differences higher than 10% were clinically significant for QoL assessment. For the rest of the variables, statistical significance was predetermined to be present at a *p*-value <0.05 on a two-sided test. Progression free survival (PFS) was determined from the first day of chemotherapy until disease progression, death, or loss of follow-up; while overall survival (OS) from starting therapy to death or last contact. PFS and OS were analyzed by the Kaplan-Meier method; the log-rank test was used to evaluate differences among subgroups. All statistical analyses were carried out using the SPSS (Statistical Package for the Social Sciences) version 26.0 (SPSS Inc, Chicago, IL, USA).

### Cost Analysis

The economic analysis that we performed followed the recommendations and guidelines for Conducting Economic Evaluations Studies in Mexico (32) and ISPOR Guidelines (33). Specifically, we conducted a cost-minimization analysis according to the results of the number needed to treat (NNT) analysis that compared four treatments that have proven to be equivalent in terms of PFS and OS (34). Assuming that the compared treatments (CIGC, SIGC, CP, and CPB) are equivalent in terms of efficacy (20, 27), this evaluation aimed to demonstrate economic benefit (savings), given the lower cost and decreased occurrence of adverse events (neutropenia and thrombocytopenia grade ≥3) with CIGC compared to the three other treatment options (SIGC, CP, CPB). Our economic analysis was carried out at a public hospital setting (INCan) as well as in a private hospital setting in Mexico. For this economic evaluation, a time horizon of six treatment cycles was considered to capture the use of resources associated with patients’ treatment (35). Only direct medical costs related to each intervention were evaluated. We considered the treatment cost in the public sector as a base case and the private sector cost as an additional case. We did not use a discount rate since the evaluated time horizon was less than 1 year. All costs were expressed in US dollars (1 USD = 21.01 MXN).

Costs of treatment were prospectively collected; cost estimation was used to adjust for the variability of treatment cost along the years (over a decade) in patients treated at our institution; owing to the single-arm design of our study, we also used cost estimation to evaluate the costs associated with other therapies. Chemotherapy costs were estimated for first-line chemotherapy regimens recommended by the National Comprehensive Cancer Network Clinical Practice Guidelines in Oncology (NCCN Guidelines) for MPM (24). Chemotherapy regimens considered were:

A). Pemetrexed 500 mg/m^2^ on day 1 plus Cisplatin 75 mg/m^2^ on day 1, administered every 3 weeks (CP)
B). Pemetrexed 500 mg/m^2^ on day 1 plus Cisplatin 75 mg/m^2^ on day 1, administered every 3 weeks, plus, bevacizumab 15 mg/kg administered on day 1 every 3 weeks (CPB)
C). Cisplatin 75 mg/m^2^ plus Gemcitabine 1,200 mg/m^2^ in a short infusion on days 1, 8, and 15 every 3 weeks (SIGC)

In the public setting, we calculated the chemotherapy cost using the wholesale acquisition cost (WAC) obtained from institutional sales portals of the Health Sector in Mexico (30). In the private setting, the cost of the pharmacological treatment was obtained from private drug-sale portals.

Treatment costs were obtained by considering each individual patient body surface area (BSA), cost per milligram, and the number of chemotherapy cycles. The total cost of the six cycles for each chemotherapy regimen resulted from the sum of every chemotherapy cycle. We did the same process for public and private settings analyses.

To obtain costs for administrating chemotherapy in public and private health settings, we used the INCan recovery fee board (USD18.13 per day) and fees from a private health care hospital (USD 38.08 per hour). The cost per minute was calculated multiplying by the number of minutes per session for each chemotherapy scheme (CGIC: 390 min, SGIC: 90, BCP: 130 min [first session] and 100 min [second cycle and beyond] and CP scheme: 70 min). We got the final six cycle cost of administration by multiplying the cost per session times the number of sessions for each cycle.

Our patients received pharmacological treatment every time they presented neutropenia grade ≥3, which was managed with antibiotics (third-generation cephalosporin) and G-CSF (filgrastim); it should be noted that none of the patients that developed thrombocytopenia required transfusion or hospitalization, therefore, only the cost of treating neutropenia ≥3 was considered. The cost of treating chemotherapy associated AE was estimated considering the resources employed and registered at the clinical chart.

We performed a univariate deterministic sensitivity analysis for cost-minimization analysis, including the results with the main parameters variation to assess the robustness of our results by modifying the variables with the greatest uncertainty in a fixed range. The variables included in the analysis were: number of cycles (3, 6, 9, and 12 cycles), treatment dose, the average weight of the patients (64.19, 63.38, and 65 kg), cost of treating neutropenia (USD797.00 and USD8,897.00), and cost of thrombocytopenia care: USD797.00 and USD8,262.00.

## Results

### Efficacy, Safety, and QoL

We included 80 patients from January 2009 to July 2019. Every included patient was diagnosed with stage III or IV MPM unresectable disease. The mean age at diagnosis was 60.4 years ( ± 11.5); 57 patients were males (71.3%), and 46 were current or former smokers (57.5%), asbestos exposure was present in only 46.2%. Epithelioid subtype was the most frequent histology, which was identified in 69 patients (86.2%). Other baseline characteristics of our population are presented at **Table 1**.

**Table 1:** Baseline characteristics of patients.

Gender		% (n); N = 80
Male		71.3 (57)
Female		28.7 (23)
**Age**		
<60 years ≥60 years		43.8 (35)
		56.2 (45)
**Smoking history**		57.5 (46)
**Asbestos exposure**		46.2 (37)
**Wood-smoke exposure**		31.2 (25)
**ECOG-PS**		
0–1 2–3		76.2 (61)
		23.8 (19)
**Histology**		
Epithelioid Other		86.2 (69) 13.8 (11)
**Karnofsky**		
<80 ≥80		65.0 (52)
		35.0 (28)
**Stage of disease**		
III IV		31.2 (25)
		68.8 (55)
**CALGB**		
1–2 3–4 5–6		25.0 (20)
		42.5 (34)
		32.5 (26)
**EORTC**		
Low risk High risk		43.8 (35)
		56.2 (45)

**Figure 1:**
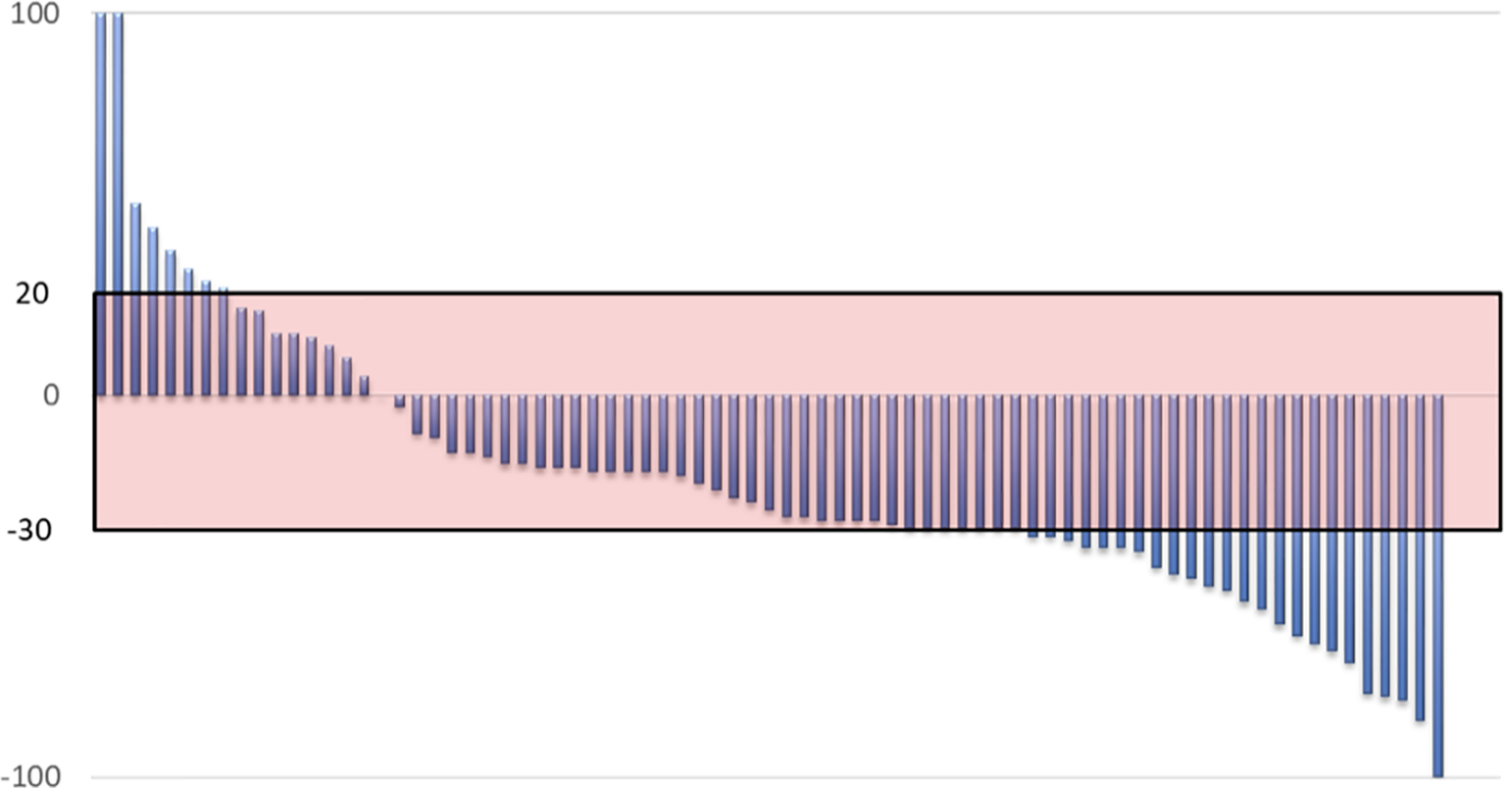
Waterfall plot representing the best response to CIGC by each individual patient.

**Figure 2:**
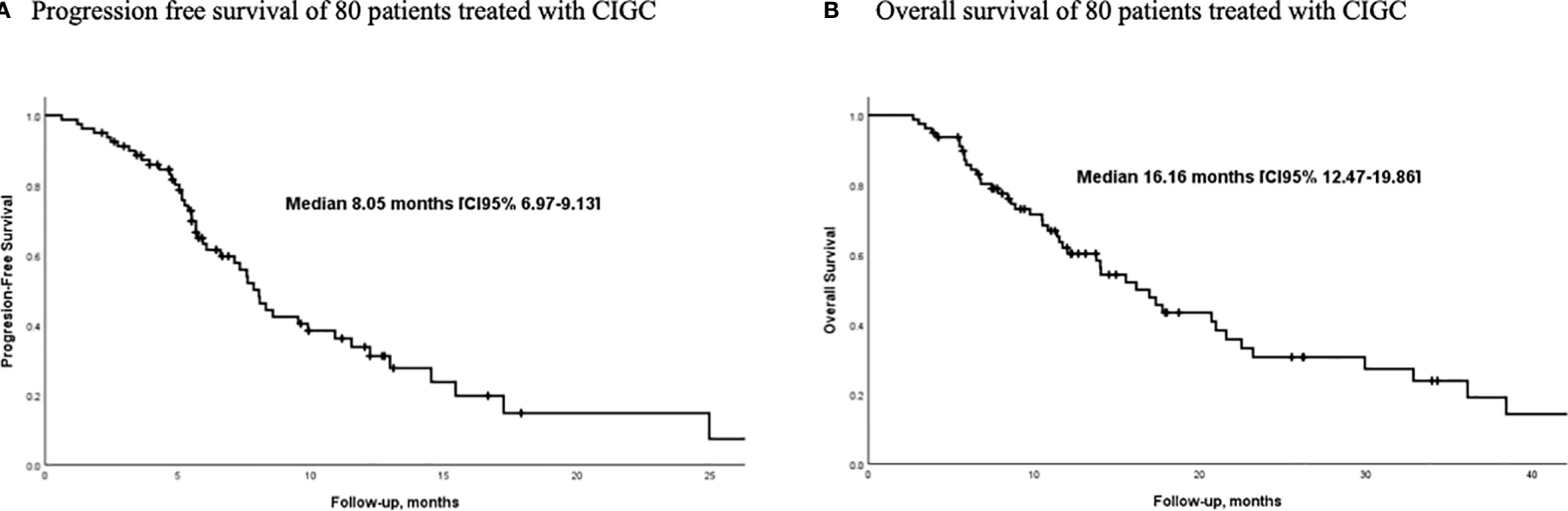
Survival outcomes of patients with MPM treated with prolonged low-dose infusion of gemcitabine and cisplatin. (n=80) **(A)** Progression-free survival **(B)** Overall survival.

The median number of chemotherapy cycles administered was 6 (range 1–11 cycles); objective response rate (ORR) (i.e., patients with partial or complete response) was documented in 46.2%, and disease control rate (i.e., patients with stable disease or partial response) was seen in 81.2%. The most common treatment response was partial response (45% of patients); just one patient achieved a complete response and continued without disease at the time of the analysis; we were unable to determine treatment response in three patients. **Figure 1** presents best treatment response for each patient displayed in a waterfall plot. Median PFS was 8.05 months (CI 95% 6.97–9.13); median OS was 16.16 months (CI 95% 12.5–19.9). **Figures 2A, B** present the Kaplan-Meier curves for PFS and OS, respectively.

**Figure 3:**
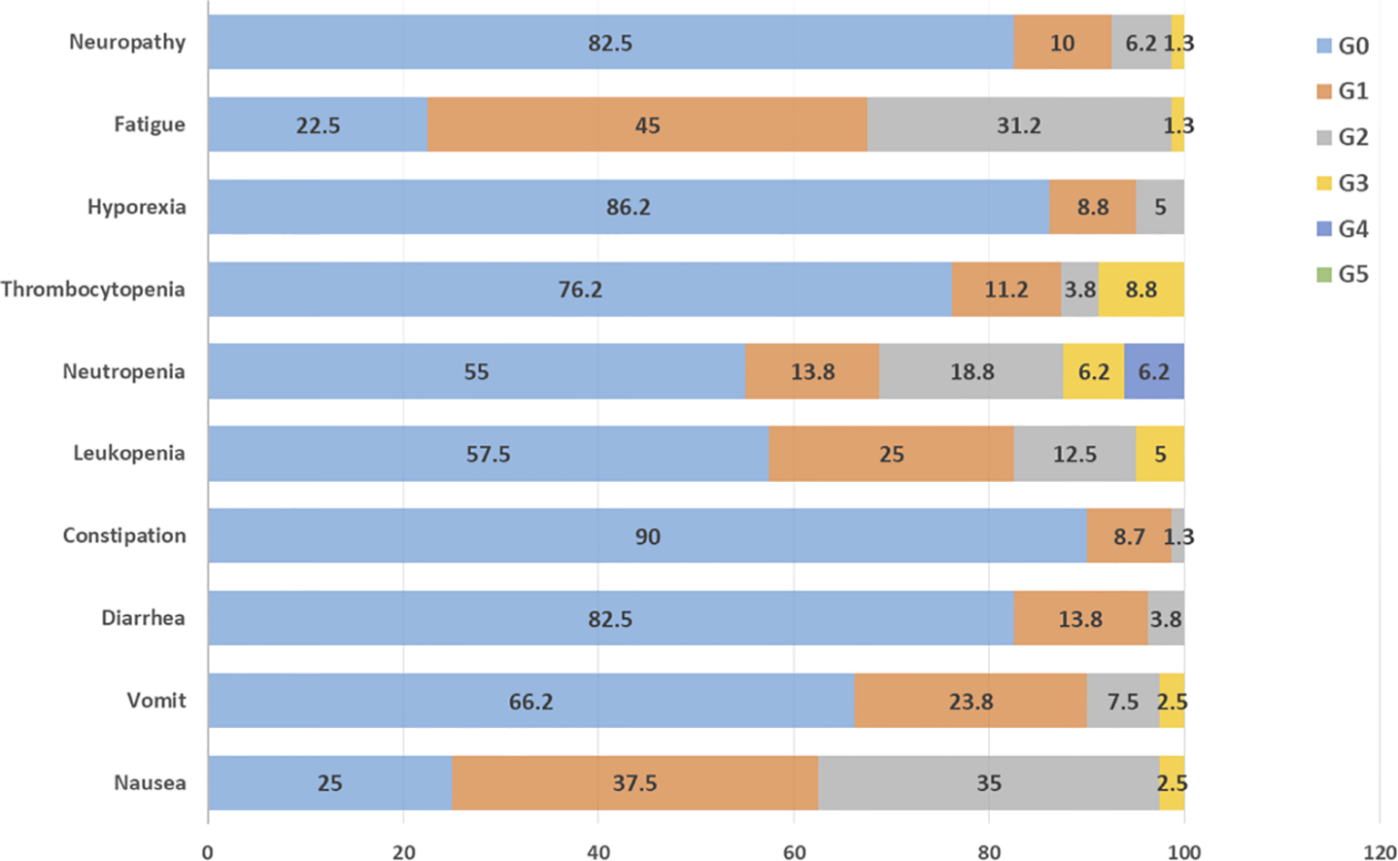
Common toxicities and respective grade (G) seen during treatment with CIGC.

Univariate analysis for baseline characteristics and PFS or OS is presented in **Tables 2** and **3**, respectively. No analyzed factors were associated with statistically significant differences in PFS. The only variables associated with significant differences in OS were gender and Karnofsky performance status. **Figure 3** presents the frequencies and grade of adverse events. The most common toxicity was fatigue (77.5%), followed by nausea (75%), leukopenia (42%), and vomit (33.8%). Severe neutropenia (grade 3–4) was observed in 10 patients (12.5%).

**Table 2:** Univariate analysis for association with progression-free survival to cisplatin/gemcitabine treatment.

	Total (events)	Median (95% CI)	p-value
**Overall**	80 (47)	8.05 (6.97–9.13)	
**Gender**			
Male	57 (32)	8.31 (5.69–10.94)	
Female	23 (15)	7.33 (5.07–9.58)	0.302
**Age, years**			
<60	35 (19)	11.53 (6.29–16.78)	
≥60	45 (28)	7.33 (5.68–8.97)	0.380
**Tobacco exposure**			
Absent	34 (17)	8.05 (5.25–10.84)	
Present	46 (30)	7.85 (6.46–9.24)	0.669
**Exposure to asbestos**			
Absent	43 (25)	8.08 (6.95–9.22)	
Present	37 (22)	7.85 (4.88–10.82)	0.648
**ECOG PS**			
0–1	61 (36)	8.05 (6.61–9.49)	
2–3	19 (11)	7.85 (4.44–11.27)	0.965
**Histology**			
Epitheloid	69 (39)	8.08 (6.92–9.25)	
Other	11 (8)	6.07 (5.26–6.89)	0.381
**Karnofsky**			
<80	28 (16)	7.12 (4.59–9.67)	
≥80	52 (31)	8.31 (5.66–10.97)	0.350
**Disease stage**			
III	25 (16)	8.05 (6.77–9.33)	
IV	55 (31)	7.85 (4.98–10.72)	0.881
**CALGB**			
1–2	20 (9)	12.22 (5.46–18.98)	
3–4	34 (19)	8.05 (6.69–9.41)	
5–6	26 (19)	6.07 (4.49–7.66)	0.682
**EORTC**			
Low risk	35 (23)	7.32 (4.51–10.14)	
High risk	45 (24)	8.08 (5.41–10.75)	0.285

**Table 3:** Univariate analysis of the factors associated with overall survival to cisplatin/gemcitabine treatment.

	Total (events)	Median (95% CI)	p-value
**Overall**	80 (46)	16.16 (12.47–19.86)	
**Gender**			
Male	57 (31)	17.35 (11.88–22.81)	
Female	23 (15)	14.03 (0.37–27.69)	**0.007**
**Age, years**			
<60	35 (20)	20.69 (11.80–29.59)	
60+	45 (26)	15.54 (9.76–21.32)	0.599
**Tobacco exposure**			
Absent	34 (23)	14.03 (8.75–19.30)	
Present	46 (23)	17.35 (9.71–24.98)	0.062
**Exposure to asbestos**			
Absent	43 (23)	15.54 (9.58–21.50)	
Present	37 (23)	17.74 (7.39–28.09)	0.330
**ECOG PS**			
0–1	61 (35)	17.35 (11.34–23.36)	
2–3	19 (11)	9.76 (7.11–12.41)	0.084
**Histology**			
Epithelial	69 (40)	16.95 (10.81–23.09)	
Other	11 (6)	13.99 (5.31–22.68)	0.369
**Karnofsky**			
<80	28 (20)	11.53 (9.33–13.73)	
80+	52 (26)	17.74 (10.93–24.75)	**0.020**
**Disease stage**			
III	25 (16)	16.95 (12.69–21.21)	
IV	55 (30)	15.54 (5.75–25.33)	0.789
**CALGB**			
1–2	20 (13)	20.69 (14.06–27.34)	
3–4	34 (19)	13.99 (6.99–20.99)	
5–6	26 (14)	11.73 (5.62–17.84)	0.436
**EORTC**			
Low risk	35 (19)	17.35 (15.24–19.46)	
High risk	45 (27)	13.77 (3.49–24.03)	0.404

P values in bold are the ones with a statistically significant value.

Regarding QoL assessments, functional role, emotional role, and pain reached a significant clinical improvement after two chemotherapy cycles. Of note, nausea/vomit symptoms significantly increased after two cycles of chemotherapy (**Supplementary Figures 1A**).

### Cost-Minimization Analysis Results

The cost of acquiring each individual drug, cost of in-patient chemotherapy administration, and the cost of treating AE (neutropenia and thrombocytopenia) for each chemotherapy regimen are summarized in**Supplementary Table 1**. Of note, CIGC was the least expensive option in both the public and private settings. This remains true after pondering the price of drugs and treatment employed to relieve AEs. To compare the prices and the rate of AEs seen in SIGC, CP, and CPB, we used prior results and toxicity profiles published by other groups, as stated in **Supplementary Table 2**.

Cost-minimization analysis was calculated separately from the public and private healthcare settings. **Supplementary Figure 2** presents the results of the cost-minimization analysis for the public healthcare perspective. The per-milligram analysis revealed that the mean total cost of therapy was $367.57, $1,092.75, $16,020.69, and $964.56 per patient for CIGC, SIGC, CPB, and CP, respectively. From the total cost in each regimen, chemotherapy represented the most robust expenditure, which accounted for 68, 79, 99, and 89% for CIGC, SIGC, CPB, and CP groups, respectively. Likewise, savings represented 66.4, 61.9, and 97.7% comparing CIGC with SIGC, CP, and CPB, respectively.

The resources spending employed for the treatment of AEs (neutropenia and thrombocytopenia) were lower in the CIGC group, calculating for savings that would be achieved by treating a larger number of patients with the CGCI *vs.* CGSI *vs.* CPB *vs.* CP the thrifts are constant. Representing greater savings while treating more patients.

**Supplementary Figure 3** shows the results of the cost-minimization analysis for a private healthcare perspective. The per-milligram analysis revealed a mean total cost of $4,224.35, $7,888.84; $39,893.42, and $9,236.68 per patient for CIGC, SIGC, CPB, and CP respectively. CIGC, compared with SIGC, represented a 46.5% savings; compared with CPB savings were of 89.4%; finally, compared with CP, savings represented 54.3%. In general, resources used to treat AEs (neutropenia and thrombocytopenia) with the CIGC scheme were less than those for SIGC, CPB, and PC. We performed a deterministic sensitivity analysis (tornado diagram) in public and private settings to validate our results, demonstrating its robustness under uncertainty conditions (see **Supplementary Figures 2** and **3**).

## Discussion

To the best of our knowledge, this was the first study to analyze the cost-effectiveness of treatment with CIGC in patients with unresectable MPM. Of note, a previous work by our group, published in 2014, analyzed this treatment strategy in 39 patients; although this is an extended version of our prior publication, this is the first time that we report the results of cost-minimization analysis. As our results suggest, this treatment provides similar efficacy to the more frequently used schemes employing CP and CPB. The non-randomized, single-arm design of our protocol is a considerable limitation to compare our results to those reported by other groups. However, due to the low incidence of MPM, we consider that results derived from this cohort (80 patients) should be enough to conclude that our treatment efficacy is similar to other strategies approved by FDA (CP or CPB) for patients with unresectable MPM.

In our population, a relatively low percentage of patients reported asbestos exposure; this percentage might be underestimated due to recall bias, which is of particular concern given the considerable latency period between asbestos exposure and MPM diagnosis. Similar to the results reported by other groups, male gender was more prevalent, and epithelioid histology was the most common. However, contrasting with other group results, we did not observe any association between the epithelioid histology subtype and PFS or OS (36, 37).

The EMPHACIS trial was the pivotal study that led to the FDA approval of PC as first-line therapy for patients with unresectable MPM; this study reported an ORR of 41.3% and a median PFS and OS of 5.7 and 12.1 months (21). Our trial showed similar ORR (46.2%), noteworthy our trial demonstrated a slightly better PFS and OS (8.5 months and 16.16 months, respectively.

The MAPS study was a randomized phase III trial that enrolled 448 patients to receive either PC or PCB as first-line therapy for advanced or metastatic MPM. The PCB regimen significantly improved OS when compared to PC regimen (18.8 months *vs.* 16 months) at the cost of increased manageable adverse events (20). According to these results, this treatment should be considered a suitable option for patients who can afford bevacizumab despite the non-negligible costs pemetrexed itself represents.

In a multicenter, randomized phase II trial published by Kindler et al., 106 chemotherapy-naive patients were treated with CG with or without bevacizumab; notably, no OS benefit (15 months in both arms) was observed with the addition of bevacizumab (38). However, subsequent therapy lines, like the use of pemetrexed, might obscure the potential antiangiogenic benefits. It should be pointed out that Kindler *et al.* administered a higher dose of gemcitabine (1,250 mg/m2) on days 1 and 8 of a 21-day cycle; furthermore, gemcitabine was infused in a short period (38). Therefore, whether bevacizumab could improve oncological outcomes in the context of based low dose gemcitabine continuous infusion should be further evaluated, ideally in a more extensive randomized phase III trial.

QoL assessment results reflected every functional scale improvement after two chemotherapy cycles, with the emotional and functional role reaching a clinically significant (≥10%) improvement. Likewise, pain and dyspnea reached a statistical and clinically significant improvement at QoL reassessment. Contrariwise, nausea and vomit were the only symptoms that were significantly worse after two chemotherapy cycles. These results are aligned with those reported by others and highlight the importance of chemotherapy to improve QoL (10, 39). The fact that we evaluated QoL after just two chemotherapy cycles should be consider a limitation of our study, this is especially important if we attempt to compare our results with those reported by other groups, which generally evaluate QoL modifications after more chemotherapy cycles have been administered; as mentioned earlier (methods subtitle), owing to the intrinsic characteristics of our population, evaluating QoL after just two cycles was more convenient in our trial.

The most common risk factor for developing MPM has been linked to low-socioeconomic status (1). Therefore, this situation often imposes a monetary access barrier for patients who cannot afford a pemetrexed based regimen. Consequently, alternative strategies, preferentially not expensive, should be pursued as possible in this subset of patients and low- and middle-income countries. The major limitation of the present study was the single-arm design without the possibility of setting direct comparisons with other treatment modalities. This is of great relevancy since the basis of cost-minimization analyses is that they are only valid under the assumption of equivalence regarding treatment outcomes between available treatment modalities; we performed the study assuming that CICG has equivalent clinical efficacy to PCB, PC, and SIGC as frontline therapy for advanced MPM. Another limitation of our cost analysis might be that we could underestimate social costs related to therapy because we did not include the costs associated with patient caregivers. Furthermore, the relatively high number of patients censored from survival analysis might be considered as a limitation; this high percentage of censored patients is explained by the characteristics of our Institution and our population. Our Institution receives patient from underserved communities all across the country, loss of follow-up is frequently seen in our patients because a lot of patients treated at our institution came from distant rural areas and are from extremely limited monetary communities. Because of this, many patients stop treatment without ever informing our physicians and continue their treatment, or die, at their local hospitals.

Finally, the recent approval of immunotherapy (Ipilimumab plus Nivolumab) as the first-line treatment for patients with unresectable MPM imposes another limitation to our study, since we did not perform a cost analysis comparing CIGC with the newly approved immunotherapy regimen. However, we consider that even if the survival benefits associated with immunotherapy used as first-line therapy are undeniable, these benefits are directly associated with an exponentially increased cost. In the particular setting of our monetary-limited population, immunotherapy might not be affordable in the foreseeable future; this assumption is definitively different in the setting of developed countries with stronger economies and more developed healthcare systems. Also, results derived from our costs analysis might differ significantly in the future as drugs such as pemetrexed and bevacizumab had recently lost their patents; accordingly, the introduction of generic and biosimilar drugs might significantly modify current costs associated with the analyzed schemes.

## Conclusion

Continuous infusion of low-dose gemcitabine plus cisplatin is an effective and safe treatment option for patients with unresectable MPM; besides, this combination is a cost-saving option when compared with SIGC, CP, or CPB. Therefore, this treatment scheme should be strongly considered for patients with unresectable MPM and limited economic resources; owing to the single arm, single center design of our study, we consider that our results should be corroborated by multicentric, randomized trials.

## Data Availability Statement

The original contributions presented in the study are included in the article/**Supplementary Material**. Further inquiries can be directed to the corresponding author.

## Ethics Statement

All patients provided written informed consent to participate, and the scientific and bioethical committees of INCan approved the entire study. The patients/participants provided their written informed consent to participate in this study.

## Author Contributions

Conception and design: all authors. Administrative support: OA, HS-M, SV, JT, and AC. Provision of study materials or patients: WM-M, RC, MR-R, LC-M, SC, FB, and OA. Collection and assembly of data: WM-M, RC, HS-M, SV, OD-Á, VM-P, and JT. Data analysis and interpretation: WM-M, SM-H, RC, HS-M, SV, VM-P, OD-Á, and OA. All authors contributed to the article and approved the submitted version.

## Conflict of Interest

OA has received honoraria as an advisor, participated in speakers’ bureau, and given expert opinions to Pfizer, AstraZeneca, Boehringer Ingelheim, Roche, Lilly, and Bristol-Myers Squibb. SM-H, SV, OD-Á, and VM-P declare that they are employees of HS Estudios Farmacoeconómicos S.A. de C.V., are ISPOR members, and declare have received honoraria from Roche, Novartis, Sanofi, Takeda, Pfizer, and Biogen as well as have served in a consulting or advisory role for Roche, Celgene, and AstraZeneca.

The remaining authors declare that the research was conducted in the absence of any commercial or financial relationships that could be construed as a potential conflict of interest.

## Supplementary Material

Supplementary Figure 1 Quality of life assessment prior to the first chemotherapy cycle and after two cycles.

Supplementary Figure 2 Cost minimization analysis of CIGC *vs* SIGC **(A, B,** and **C)**; CIGC *vs* CP **(D, E,** and **F)**; and CIGC *vs* CPB **(G, H,** and **I)** at a public hospital setting.

Supplementary Figure 3 Cost minimization analysis of CIGC *vs* SIGC **(A, B,** and **C)**; CIGC *vs* CP **(D, E,** and **F)**; and CIGC *vs* CPB **(G, H,** and **I)** at a private hospital setting.
