# Supplementary material for: Efficacy, Safety, and Cost-Minimization Analysis of Continuous Infusion of Low-Dose Gemcitabine Plus Cisplatin in Patients With Unresectable Malignant Pleural Mesothelioma
Source: Front Oncol. 2021 Apr 20;11:641975. doi: 10.3389/fonc.2021.641975 (PMC8095245; doi:10.3389/fonc.2021.641975)
Supplement: Supplementary file 1 [file DataSheet_1.docx]

**Supplementary Table 1. Total cost of treatment, treatment administration and treatment of AE for 6 cycles of chemotherapy. Amounts are presented in USD.**

|  | **CIGC** | **SIGC** | **CP** | **CPB** |
| --- | --- | --- | --- | --- |
| **Public hospital setting** | | | | |
| **Drug cost** | $248.97 | $865.90 | $858.14 | $15,909.36 |
| **Administration cost** | $117.87 | $22.67 | $10.58 | $15.87 |
| **Cost of treating AE**  Neutropenia G3/4  Thrombocytopenia G3/4 | $0.73  $0 | $102.78  $101.41 | $85.09  $10.76 | $82.96  $12.51 |
| **Total cost** | **$367.57** | **$1,092.76** | **$964.57** | **$16,020.70** |
| **Private hospital setting** | | | | |
| **Drug cost** | $1,209.21 | 6,119.99 | $8,372.14 | $38,899.72 |
| **Administration costs** | $2,970.01 | 571.16 | $266.54 | $399.81 |
| **Cost of treating AE**  Neutropenia G3/4  Thrombocytopenia G3/4 | $45.13  $0 | $652.45  $545.25 | $540.14  $57.86 | $526.65  $67.24 |
| **Total cost** | **$4,224.35** | **$7,888.85** | **$9,236.68** | **$39,893.42** |

**Supplementary Table 2.** Proportion of patients with AE (neutropenia and thrombocytopenia)

| **Scheme** | **AE** | **Grade 1 a 4 n(%)** | **Grade 3 y 4 n(%)** |
| --- | --- | --- | --- |
| **CIGC^1^**  (N:80) | Neutropenia  Thrombocytopenia | 12(15.1)  1(1.4) | 5 (6)  0 |
| **SIGC^2^**  (N:53) | Neutropenia  Thrombocytopenia | 40(76)  51(96) | 30(56)  26(49) |
| **CPB^3^**  (N:222) | Neutropenia  Thrombocytopenia | 173(77.9)  130(58.6) | 98(44.1)  22(9.9) |
| **CP^3^**  (N:224) | Neutropenia  Thrombocytopenia | 177(79)  119(53.1) | 100(44.6)  21(9.4) |
| ^1^Data obtained from the database of this study; ^2^AK Nowak, 2002; ^3^Zacman, 2016. | | | |
