# Supplementary material for: Efficacy, Safety, and Cost-Minimization Analysis of Continuous Infusion of Low-Dose Gemcitabine Plus Cisplatin in Patients With Unresectable Malignant Pleural Mesothelioma
Source: Front Oncol. 2021 Apr 20;11:641975. doi: 10.3389/fonc.2021.641975 (PMC8095245; doi:10.3389/fonc.2021.641975)

Supplementary Figure 1

Supp Fig. 1-A

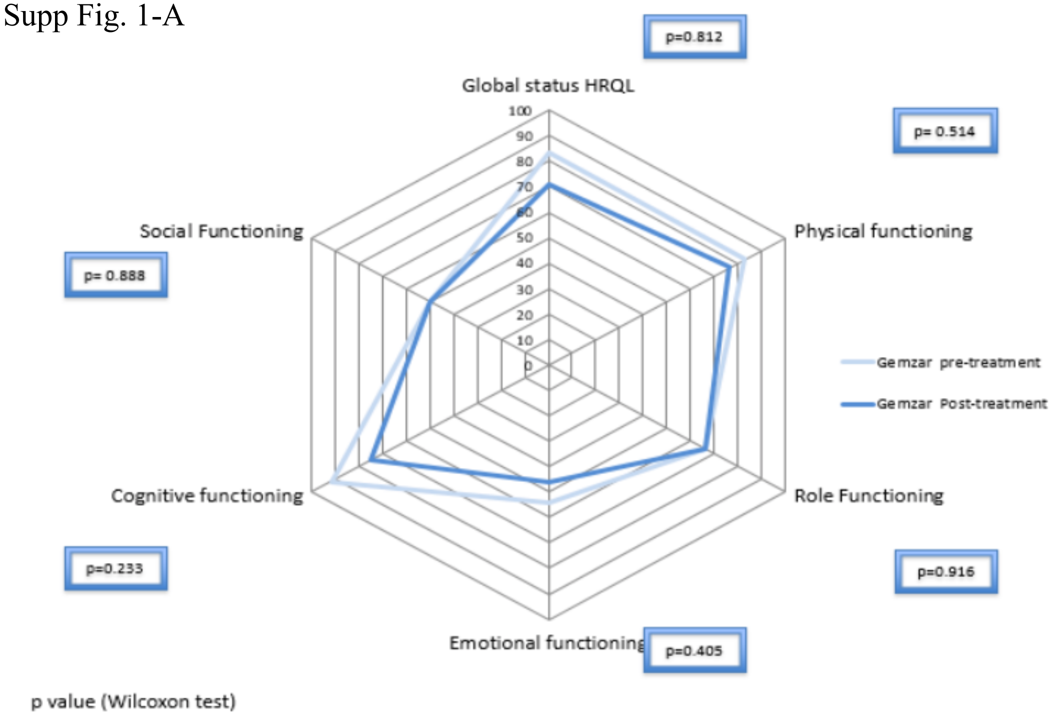

Supp Fig. 1-B

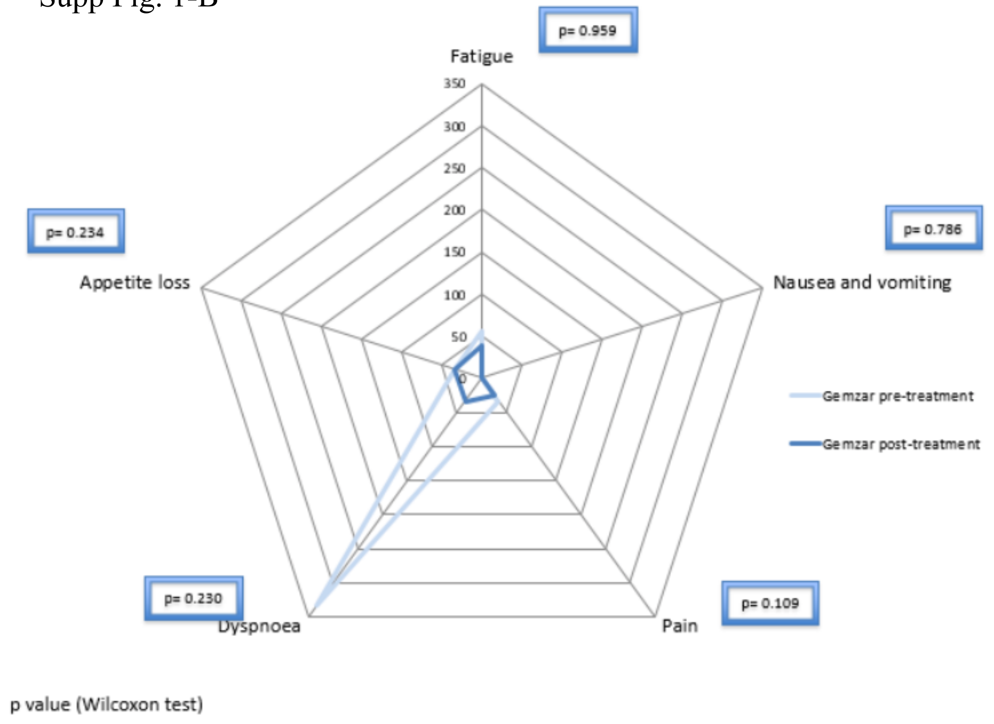

Supplementary Figure 2

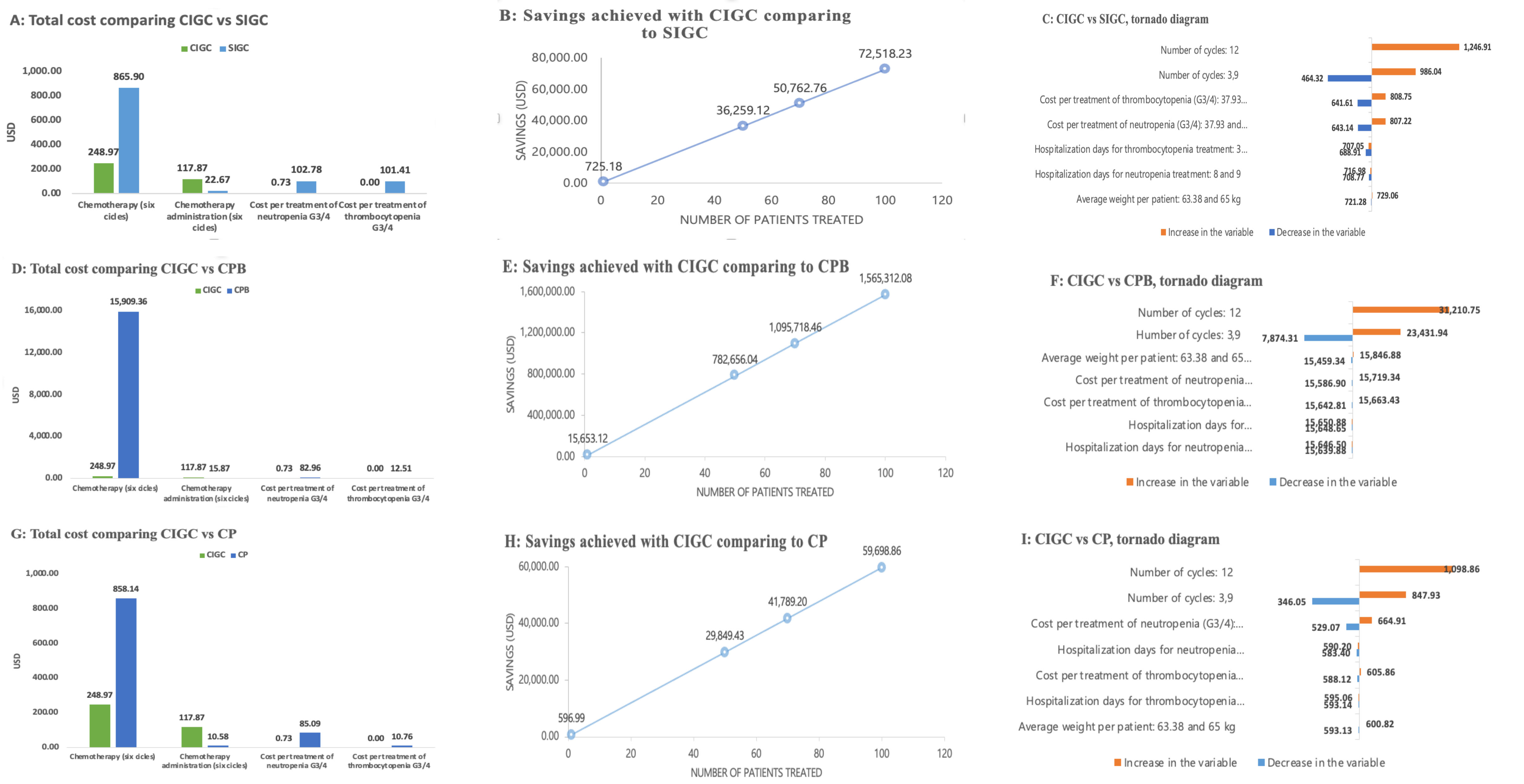

Supplementary Figure 3

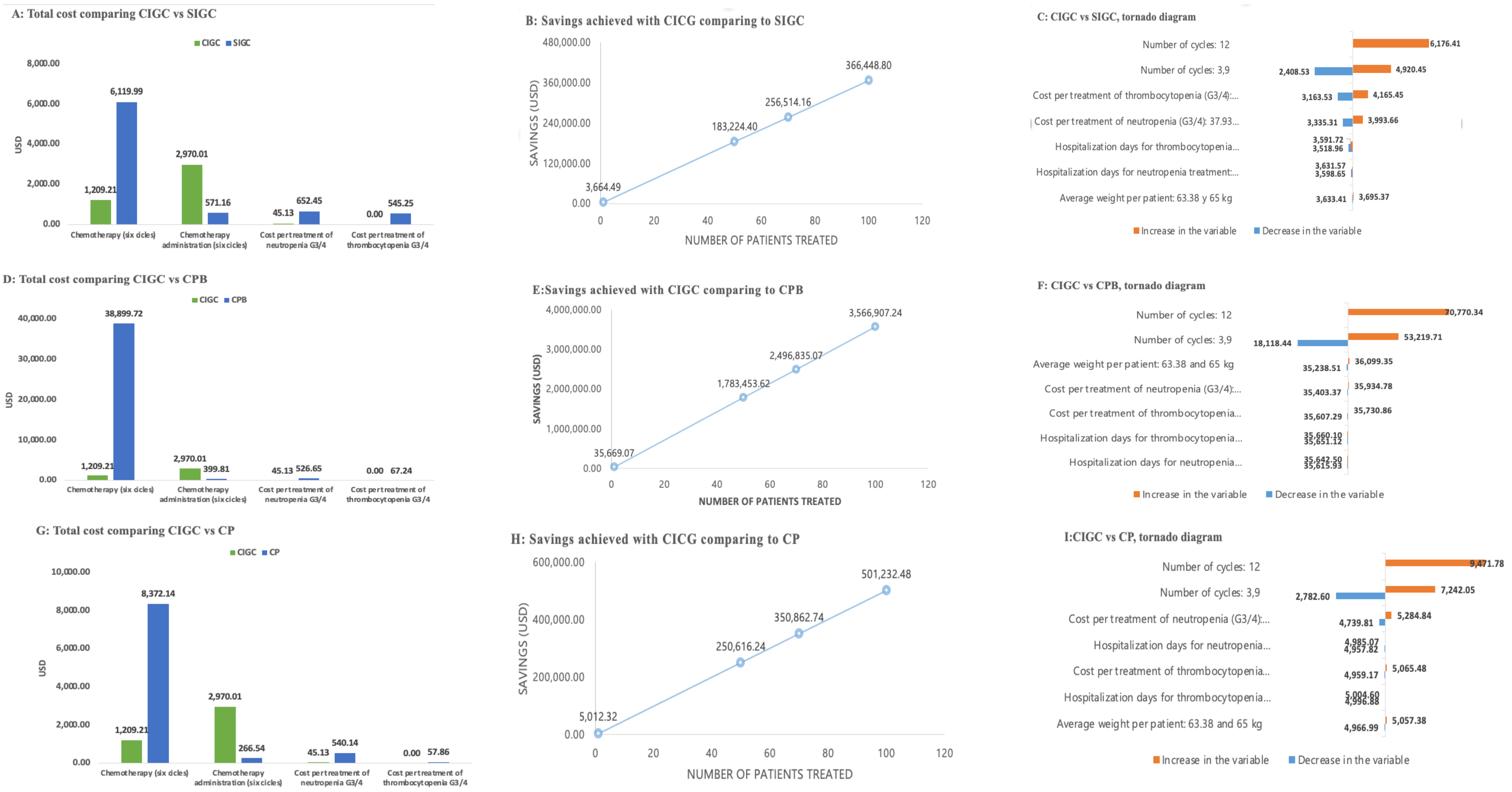

Supplement: Supplementary Figure 1 — Quality of life assessment prior to the first chemotherapy cycle and after two cycles. [file DataSheet_2.pdf]
